# Fullerol rescues the light-induced retinal damage by modulating Müller glia cell fate

**DOI:** 10.1016/j.redox.2023.102911

**Published:** 2023-10-05

**Authors:** Zhe Cha, Zhiyuan Yin, Luodan A, Lingling Ge, Junling Yang, Xiaona Huang, Hui Gao, Xia Chen, Zhou Feng, Lingyue Mo, Juncai He, Shuang Zhu, Maoru Zhao, Zui Tao, Zhanjun Gu, Haiwei Xu

**Affiliations:** a Southwest Eye Hospital, Southwest Hospital, Third Military Medical University (Army Medical University), Chongqing, 400038, China; b Key Lab of Visual Damage and Regeneration & Restoration of Chongqing, Southwest Eye Hospital, Southwest Hospital, Chongqing 400038, China; c CAS Key Laboratory for Biomedical Effects of Nanomaterials and Nanosafety and CAS Center for Excellence in Nanoscience, Institute of High Energy Physics and National Center for Nanoscience and Technology, Chinese Academy of Sciences, Beijing, 100049, China; d College of Materials Science and Optoelectronic Technology, University of Chinese Academy of Sciences, Beijing, 100049, China; e Joint Logistics Support Force of Chinese PLA, No. 927 Hospital, Puer 665000, Yunnan, China

**Keywords:** Fullerol, Light-induced retinal damage, Müller glia, De-differentiation, Retina

## Abstract

Excessive light exposure can damage photoreceptors and lead to blindness. Oxidative stress serves a key role in photo-induced retinal damage. Free radical scavengers have been proven to protect against photo-damaged retinal degeneration. Fullerol, a potent antioxidant, has the potential to protect against ultraviolet-B (UVB)-induced cornea injury by activating the endogenous stem cells. However, its effects on cell fate determination of Müller glia (MG) between gliosis and de-differentiation remain unclear. Therefore, we established a MG lineage-tracing mouse model of light-induced retinal damage to examine the therapeutic effects of fullerol. Fullerol exhibited superior protection against light-induced retinal injury compared to glutathione (GSH) and reduced oxidative stress levels, inhibited gliosis by suppressing the TGF-β pathway, and enhanced the de-differentiation of MG cells. RNA sequencing revealed that transcription candidate pathways, including Nrf2 and Wnt10a pathways, were involved in fullerol-induced neuroprotection. Fullerol-mediated transcriptional changes were validated by qPCR, Western blotting, and immunostaining using mouse retinas and human-derived Müller cell lines MIO-M1 cells, confirming that fullerol possibly modulated the Nrf2, Wnt10a, and TGF-β pathways in MG, which suppressed gliosis and promoted the de-differentiation of MG in light-induced retinal degeneration, indicating its potential in treating retinal diseases.

## Highlights

• Fullerol might be preferentially accumulated in Müller cells (MG).
• Fullerol protects against retinal damage by modulating Müller glia (MG) fate.
• Fullerol modulates gene pathways (Nrf2, Wnt10a, and TGF-β) for retinal protection.

## Graphical abstract

**Figure:**
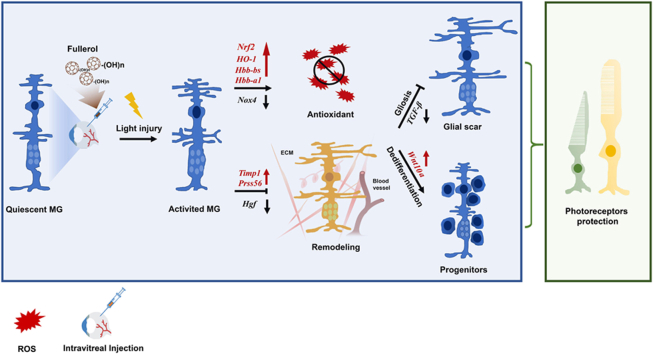

## 1 Introduction

The photoreceptors contain more mitochondria than other retinal cells and are highly light-sensitive [1,2]. Exposure to excessive light, including white light-emitting diode (LED) which emits multispectral light, can damage photoreceptors and cause visual dysfunction [3]. Light-induced retinal injury was reported to involve the processes including free radical generation, lipid peroxidation, and calcium overload [4,5]. Among these, oxidative stress is considered to exert a pivotal role in the photoreceptors damage. Prolonged light exposure usually causes the accumulation of reactive oxygen species (ROS), including oxygen free radicals, which are also linked to retinal degenerative diseases like age-related macular degeneration (AMD) and retinitis pigmentosa (RP) [6,7]. Presently used antioxidants usually focus on protecting the retina against acute phototoxicity caused by a single-spectrum light source. However, these antioxidants may not effectively prevent the long-term degeneration and loss of retinal neurons due to the overwhelming presence of ROS and the complexity of free radical species [8,9], implying that more potent free radical scavengers are required.

Fullerol is a nanomaterial with outstanding broad-spectrum antioxidant activity that can effectively remove ROS and reactive nitrogen species [10]. Therefore, it has been used to treat oxidative stress associated diseases, including neurodegenerative disorders [11] as well as radiation-induced injuries affecting the corneal, gastrointestinal system, and skin [[12], [13], [14]]. The antioxidant mechanism of fullerol involves the up-regulation of phase II antioxidant enzymes which was modulated by nuclear factor erythroid 2-related factor 2 (Nrf2), and it was confirmed in H_2_O_2_-induced apoptosis of alveolar epithelial cells [15], ischemia-reperfusion induced myocardial injury [16], and UVB-induced cornea injury [14]. Nrf2 related antioxidant enzymes, including γ-glutamate cysteine ligase, NAD(P)H: quinine oxidoreductase 1, along with heme oxygenase-1 (HO-1) were involved in the effect of fullerol [[14], [15], [16]]. Under oxidative stress, Nrf2 was observed to translocate to the nucleus where it bound to a DNA promoter and initiated transcription of antioxidative genes [17,18]. Clinically, Nrf2 activator hydroxylamine has been applied to treat AMD patients and delayed the progression of visual loss [19]. Recently, Nrf2 has also been regarded as a key sensor and effector to prevent retinal gliosis [20]. Additionally, fullerol effectively promoted stem cells to differentiate into osteoblasts through FoxO1 signaling, except for its anti-oxidative stress in bone injury [21]. Our previous results demonstrated that fullerol stimulated the proliferation of limbal stem cells in UVB-exposed corneas [14]. It suggests that fullerol alleviates oxidative damage and potentially activates endogenous stem cells to regenerate and repair damaged tissues.

Müller glia (MG) cells, the primary glial cells in the neural retina, play an important role in retinal redox homeostasis [22,23]. MG cells release cytoprotective antioxidants glutathione (GSH) and upregulate the expression of Nrf2, including catalase, glutathione peroxidase, superoxide dismutase 2, and heme oxygenase, to mitigate ROS and alleviate neuronal death [24,25]. In zebrafish, MG cells also serve as a source of endogenous stem cells for regenerating the retina after injury [22,23]. However, mammalian MG cells have limited regenerative potential to generate stem or progenitor cells [26,27]. Disruption of redox homeostasis due to photooxidative injury in mammals triggers MG activation and persistent gliosis, leading to an imbalance in the internal retinal environment, progressive visual impairment and subsequent vision loss [21,28,29].

During damage and degeneration, MG cells exhibit limited resistance to oxidative stress [30,31]. The failure of mammalian MG reprogramming may be related to an imbalance in redox homeostasis [[31], [32], [33]]. As fullerol is characterized with potent anti-oxidative stress ability and can activate endogenous stem cells, we speculate that fullerol might reverse the fate of MG gliosis, thereby protecting photoreceptors in light-induced retinal injury. In the present study, we used a Müller cell lineage-tracing mouse model and cyanine-5 (Cy5)-labeled fullerol to demonstrate that fullerol was predominantly accumulated in MG cells, thereby inhibiting gliosis while promoting the proliferation of MG cells. These effects had a notable positive impact on visual function in photodamaged mouse retinas. Using GSH as a positive control, we demonstrated that the antioxidant effect of fullerol was better than that of GSH. Therefore, the protective effect of fullerol was achieved both by the anti-oxidative effect and its ability to redirect MG cells from gliosis to de-differentiation. This process may be regulated by the Nrf2/Wnt10/TGF-β pathways. Using the H_2_O_2_-treated human MG cell line, MIO-M1, we confirmed the involvement of the Nrf2 pathway in the antioxidant activity of fullerol.

## 2 Materials and methods

### 2.1 Animals

8-week-old male and female C57BL/6J mice were provided by the Experimental Animal Center of Army Medical University, Chongqing, China. Glast-CreER transgenic mice crossed with the Cre-inducible CAG-LSL-tdTomato reporter [B6/JNju-H11^em1Cin(CAG−LoxP-ZsGreen-Stop-LoxP-tdTomato)^/Nju] were purchased from Jackson Laboratory (JAX Mice Service, stock no. 012586). Fluorescent staining of Sox9, a commonly used MG marker, was performed to confirm the validity of the MG lineage mice (Fig. S1). All of the mice were housed in a pathogen-free environment at 55 ± 5% humidity, 25 °C, and a 12:12 h light/dark cycle. Sterile water and standard chow were fed an ad libitum diet. All animal experiments were carried out as per the guidelines of the University Animal Ethics Committee and were authorized via the Laboratory Animal Management and Use Committee of the Army Medical University. The animal experiments were conducted in accordance with the guidelines of the Association for Research in Vision and Ophthalmology (ARVO). The Institutional Animal Care and Use Committee (approval number AMUWEC20182139) approved the experimental animal protocol at Army Military Medical University.

### 2.2 Drugs

H_2_O_2_ and GSH solutions were purchased from Sigma-Aldrich (St. Louis, MO, USA) and Beyotime Biotechnology (Shanghai, China), respectively. As vitamin E is fat-soluble and does not compare well with fullerol, we used water-soluble antioxidant in ophthalmic clinical practice, GSH, as a positive control in this study. GSH was solubilized in phosphate-buffered saline (PBS) to an equivalent molar concentration (94.34 μM) as fullerol. Fullerol was prepared according to a previous study [13] and was firstly dissolved in ultrapure water, then prepared to 100 μg/ml in PBS. Fullerol was labeled with the red fluorophore Cy5 to trace its distribution in the cells/retinas. Fullerol/GSH was injected into the vitreous space 45 min before light exposure. Mice were administered a single intravitreal injection of 1 μL of fullerol/Cy5-fullerol (100 μg/ml) or GSH (94.34 μM) in the left eye; the right eye was injected with PBS (1 μL) as a control. Tamoxifen (120 mg/kg body weight; Sigma, T5648) was solubilized in corn oil and administered intraperitoneally for five consecutive days to induce tdTomato fluorescence in mice.

### 2.3 Cell culture

MIO-M1, a human MG cell line, was kindly gifted by Professor Jinfa Zhang of Shanghai Jiao Tong University, Shanghai, China. MIO-M1cells were cultured in Dulbecco's modified Eagle's medium (Gibco, Grand Island, NY, USA) with 10% fetal bovine serum (FBS; Beyotime) and 1% penicillin-streptomycin (Beyotime, Shanghai, China). These cells were incubated under humidified conditions (with 5% CO_2_) at 37 °C. Cells in the fullerol-treated group were first incubated in medium containing 100 μg/ml fullerol for 2 h, followed by exposure to hydrogen peroxide (H_2_O_2_) for 24 h. To track the biodistribution of Cy5-fullerol in cells, we co-cultured the cells with Cy5-fullerol for 24 or 72 h. The GSH-treated cells were utilized as a positive control.

### 2.4 Cell cytotoxicity assay

Cell viability was identified with CCK-8 (Dojindo, Shanghai, China). MIO-M1 cells (1 × 10^4^/per well) were transferred into plates (96-well) for 24 h and later cultivated with concentration gradients of H_2_O_2_ (100, 300, 600, 1000, and 2000 μM) or fullerol (10, 50, 100, 200, and 500 μg/ml) for 24/26 h. Oxidative stress was induced using 100 μM H_2_O_2_. After treatment of cells for 24 h under the conditions mentioned, the medium was aspirated and the cells were rinsed utilizing PBS, later 10 μL/well CCK-8 solution was added to the wells with fresh medium free of FBS and cultured for 2 h. Measurement of absorbance was implemented at 450 nm by applying a microplate reader (Infinite F50, Switzerland).

### 2.5 Light exposure

**Fig. 1:**
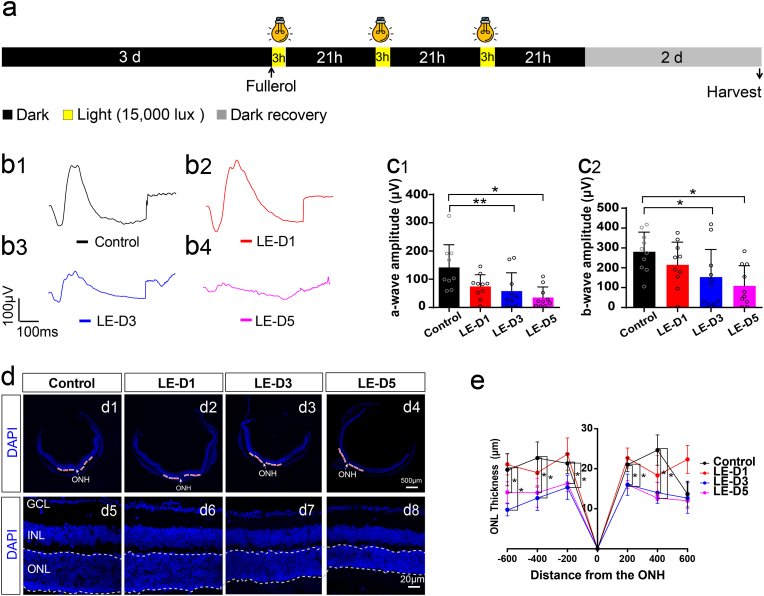
Light damage reduces the number of photoreceptors and impairs retinal responsiveness. (a) Experimental protocol and timeline of light-induced retinal degeneration model. (b) Representative scotopic ERGs response to 3.0 log cd s/m^2^ flashes curves for the a- and b-waves. (c) Illumination irradiation decreases both the a- and b-waves in the ERGs. (d) Typical images showing DAPI staining of retinal sections (Control, several time points after photodamage). (e) The ONL thickness was measured at temporal and nasal sides at 200, 400, and 600 μm from the optic nerve head and at different time points after light exposure. LE-D1, light exposure for one day; LE-D3, light exposure for three days; LE-D5, light exposure for five days. ONL, outer nuclear layer; INL, inner nuclear layer; GCL, ganglion cell layer. ONH, optic nerve head. A White dotted line indicates ONL. The data are shown as the mean ± SD; n = 4 or 9. *, P < 0.05; **, P < 0.01. Scale bars, 500 or 20 μm.

The parameters of the illumination model were established according to previous reports, with practical improvements [34,35]. We used light-emitting diodes (Xanlite XXX Evolution, France) with a 400–700 nm wavelength range in the experiments. The control group was maintained in the dark throughout the experiment. Light-exposed experiments were carried out in a homemade cage with reflective inner surfaces and an average white light luminance of 15,000 lux. Mice were dark-adapted for 72 h, followed by pupillary dilation prior to light irradiation. Mice were then exposed for 1, 3, and 5 d (3 h/d), respectively, in 15,000 lux to determine the duration of exposure needed to damage the retina. After each light exposure, the mice were kept in darkness for 21 h and finally allowed to recover for 2 d in darkness before electroretinography or histological analysis. Finally, a 3-d light exposure period was selected for subsequent experiments (Fig. 1a).

### 2.6 Outer nuclear layer thickness analysis

**Fig. 2:**
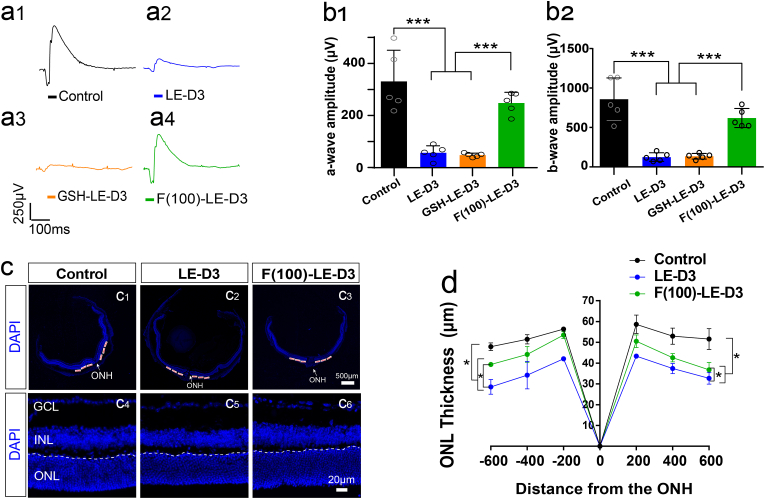
Effect of fullerol pre-treatment on light-induced retinal degeneration in mice. (a) Different groups' representative a-wave and b-wave waveforms (response to 3.0 log cd s/m^2^ flashes curves). (b) Amplitudes of a- and b-waves of the ERGs at 3 days after light exposure with 100 μg/ml concentration of fullerol. Light exposure decreases both the a- and b-waves, and prior treatment to fullerol attenuated the reduction in the ERGs. (c) Typical images showing DAPI staining of retinal sections (control, light-damaged group, fullerol-treated groups). (d) The ONL thickness was measured at temporal and nasal sides at 200, 400, and 600 μm from the optic nerve head and at different concentrations of fullerol after light exposure. F (100)-LE-D3, three days of light exposure after pretreatment with 100 μg/ml fullerol. GSH-LE-D3, three days of light exposure after pretreatment with 94.34 μM concentration of GSH. ONL, outer nuclear layer; INL, inner nuclear layer; GCL, ganglion cell layer. ONH, optic nerve head. A White dotted line indicates ONL. Data are the means ± SD; n = 4 or 5. *, P < 0.05; ***, P < 0.001. Scale bars, 500 or 20 μm.

The thickness of retinal outer nuclear layer (ONL) was examined via 4,6-diamidino-2-phenylindole (DAPI) staining and measured at 200, 400, and 600 μm away from the temporal to nasal side of the retina from the optic nerve head (Fig. 1, Fig. 2c). The distance and thickness of the retina were quantitated adopting ImageJ software 1.42 (National Institutes of Health, Bethesda, MD, USA).

### 2.7 Immunofluorescence staining

The mice were euthanized, and the eyes were promptly enucleated and fixed in 4% paraformaldehyde (PFA) at 4 °C for 20 min. Subsequently, the lens and cornea were removed. After fixing in 4% PFA for 90 min, the optic cups were immersed in 30% sucrose at 4 °C overnight. Following drying, they were exposed to Tissue-Tek OCT Compound (Sakura Fintek, Torrance, CA, USA), frozen, and lastly cryosected into sections of 12 μm thick. Sections were rinsed in PBS and permeabilized utilizing 0.5% Triton X-100 at 25 °C for 10 min, which were later blocked with 0.3% Triton X-100/5% bovine serum albumin (BSA) in PBS for an hour at 37 °C. The primary antibodies (Table 1) were applied diluted with the blocking solution and were cultivated at 4 °C overnight. After rinsing in PBS, secondary antibodies were added (Table 1) and cultivated at 37 °C for an hour. At last, the nuclei were counterstained with DAPI.

**Table 1:** Summary of primary and secondary antibodies.

Antibody name	Source	Catalog number	Dilution ratio
Glial fibrillary acidic protein (GFAP)	DAKO	Z033401	1:500 (IF); 1:1000 (WB)
Glutamine synthetase (GS)	ABclonal	A19641	1:500 (IF); 1:1000 (WB)
β-actin	Immunoway	YT0099	1:1000 (WB)
α-tubulin	Beyotime	AF0001	1:1000(WB)
Sox9	Immunoway/Abcam	YM6546/ab185966	1:500 (IF)
Iba1	Wako	019-19741	1:400(IF)
CyclinD1	ABclonal	A19038	1:200 (IF)
Chx10	Santa Cruz	SC-373151	1:500(IF)
Nrf2	Immunoway	YT3189	1:200 (IF)/1:1000 (WB)
Wnt10a	Immunoway	YN0283	1:500 (WB)
Heme Oxygenase1 (HO-1)	Proteintech	10701-1-AP	1:50 (IF)
Heme Oxygenase1 (HO-1)	ABclonal	A19062	1:1000 (WB)
488 donkey-mouse	Invitrogen	A21202	1:500
568 donkey-rabbit	Invitrogen	A10042	1:500
488 donkey-rabbit	Invitrogen	A21206	1:500
HRP-Goat anti-rabbit	Beyotime	A0208	1:2000
HRP-Goat anti-mouse	Beyotime	A0216	1:2000

MIO-M1 cells were inoculated onto coverslips and incubated for 24 h. Medium was aspirated following drug treatment and the cells were rinsed with PBS. Cells were later fixed with 4% PFA and rinsed thrice utilizing PBS. The remaining steps were consistent with those for retinal immunostaining. Fluorescence images were taken with confocal microscope (Zeiss LSM800; Oberkochen, Germany).

### 2.8 Electroretinography analysis

The electroretinograms (ERG) were recorded 2 d post-exposure. The electroretinographic activity was evaluated according to established methods [36]. Prior to electrophysiological testing, mice were acclimated overnight in the dark, and all experiments were conducted under dim red light. The mice were anesthetized with 1% sodium pentobarbital, and the pupils were dilated with 1% tropicamide. A clip-on contact lens electrode and reference electrode were placed on the cornea and the tongue, respectively, to receive ERG signals. The a- and b-wave amplitudes were recorded at different light stimulus intensities. We used Stimulator LS-100/200 (Mayo Corporation, Aichi, Japan) and Reti-Scan System (Roland, Germany) as electrophysiological equipment in these tests. The data proposed in this study were spot joint rod-cone responses of 3.0 cd s m^2^. Data and waveforms were analysed and plotted using GraphPad Prism 8.0 (GraphPad, USA).

### 2.9 Glutamine synthetase (GS) activity assay

GS assay kit from the Nanjing Jiancheng Bioengineering Institute (Nanjing, China) was exploited for identifying the activity of GS. The complex formed under acidic conditions exhibits a maximum absorption peak at 540 nm, which was measured using a spectrophotometer (Infinite F50, Switzerland) to determine GS activity.

### 2.10 Western blot (WB) analysis

Mice were euthanized in a CO_2_ chamber and then placed onto a surgical pad. After carefully removing the skin covering on the eyes, the eyeballs were squeezed out by pressing gently the both sides of the eyes with curved tweezers. The eyeballs were then cut off from the eye sockets and transferred in a 60 mm dish filled with PBS. Under an anatomical microscope, the cornea and lens were cut off along the corneal limbus and were carefully removed. From the remaining posterior eyecup, the neural retinas were separated gently from the underlying retinal pigment epithelium. The neuroretinas were then transferred in a freezing tube for extraction of total tissue protein. Each sample was pooled by three retinas from three mice.

The neuroretinas were suspended in protein lysis buffer consisting of 1% proteinase inhibitors. Cells were rinsed in ice-cold PBS for two times and subsequently lysed in buffer with 1% protease inhibitors. Aliquots of total proteins were isolated on SDS-PAGE precast gels (Nanjing ACE Biotechnology) for 20 min at 160 V, followed by rapid transfer to a PVDF membrane at 400 mA for 25 min. Following a 20-min block with rapid blocking buffer at 25 °C, membranes were cultured overnight at 4 °C with a variety of primary antibodies (Table 1), and the next day were rinsed in TBST (TBS with 0.1% Tween-20) thrice before applying the secondary antibodies (Table 1) and culturing at 25 °C for 1 h. An enhanced chemiluminescence (ECL) luminescence kit (Mengbio, Chongqing, China) was applied for detecting the protein band signals, which were imaged using a ChemiDoc Imaging system (Bio-Rad, California, USA). Finally, for each band, the grey value was examined using the ImageJ software (1.42, National Institutes of Health, USA)). The intact images of WB bands are shown in Fig. S6.

### 2.11 Whole transcriptome RNA-Seq analysis

C57BL/6J mice were categorized into control, light-damaged, and fullerol-treated groups. Each set of samples was pooled by three retinas from three mice. Three bio-replicates were applied to each treatment group. RNA-seq analysis was carried out at Majorbio Bio-Pharm Technology Co., Ltd. (Shanghai, China). The Illumina NovaSeq platform (NEB, USA) was used to build sequencing libraries. Retinal mRNAs were separated with oligo (dT) beads in accordance with the poly (A) selection methodology and subsequently fragmented using a fragmentation buffer. Generation of double-stranded cDNA via adopting random hexamer primers (Illumina). Phosphorylation, end repair, as well as addition of poly(A) tails were performed on purified double-stranded cDNA. Target cDNA fragments (300 bp) from the libraries were selected on 2% Low Range Ultra Agarose (Illumina, San Diego, USA). The cDNA library was enriched using PCR. The sequencing of paired-end RNA-seq libraries was conducted via applying the Illumina HiSeq xten/NovaSeq 6000 sequencer (Illumina, San Diego, CA, USA) (with a read length of 2 × 150 bp). Differential gene expression among the samples was established using DESeq2 (P < 0.05, |fold change| > 1.5). Kyoto Encyclopedia of Genes and Genomes (KEGG) pathway analysis together with Gene Ontology (GO) functional enrichment were implemented utilizing the Goatools software (http://gitub. com/tanghaibao/Goatools).

### 2.12 Quantitative polymerase chain reaction (qPCR)

For analyzing the mRNA levels of target genes, total RNA was separated from the retinas of three mice from various groups as per the instructions of the manufacturer utilizing TRIzol reagent (TaKaRa, Japan). Afterwards, cDNA was produced from RNA by applying the PrimerScript RT reagent kit and a gDNA eraser (TaKaRa, Japan). Table 2 shows the gene-specific primer pair sequences. We used TB Green to detect the amplification products of the cDNA samples using a Real-Time PCR Detection System (CTF96; Bio-Rad, USA). The relative expression levels of target genes relative to the control gene (β-actin) were calculated with 2^−ΔΔCT^ approach.

**Table 2:** Primer sequences.

Gene name	Forward 5′-3′	Reverse 5′-3′
TGF-β1	TGGAGCAACATGTGGAACTC	GTCAGCAGCCGGTTACCA
Smad1	GGCGACATATTGGGAAAGGA	TCACTGAGGCATTCCGCATA
Smad2	TCACTGAGGCATTCCGCATA	AAGCCATCACCACTCAGAATTG
Smad3	CACTGATCTACCGTATTTGCTGT	CACGCAGAACGTGAACACC
Smad4	GGCAGTAGATAACGTGAGGGA	ACACCAACAAGTAACGATGCC
β-actin	GCAAAGGTTTCACTTTCCCCA	AAGTCCCTCACCCTCCCAAAAG
Wnt10a	GGTCAGCACCCAACGACATCC	TGGCGTAGGCGAAAGCACTCT
Timp1	AGTCCCAGAACCGCAGTGAA	GTGGCAGGCAAGCAAAGTGA
HGF	CAAATGCAAGGACCTTAGAG	CCAGAAGATATGACGGTGTAA
Hbb-bs	AAAGGTGAACGCCGATGAAG	ATGATAGCAGAGGCAGAGGATAG
Hba-a1	CAGGTCAAGGGTCACGGCAAGA	GGGTGAAATCGGCAGGGTGG
Hmox1	TGACAGAAGAGGCTAAGACCG	AGTGAGGACCCACTGGAGGA
Serpine3	ACGGCACAACTCCAGTCAAG	CCTCCGTGGTAGTGCTGTTAG
Nox4	TCAAACAGATGGGATTCAGA	GAGTTGTTCCGGTTACTCAA
Prss56	GAGGCTGCAACTTGGAGGGT	GTCTGCGGGTCAAACTTAGGG

### 2.13 Statistical analysis

GraphPad Prism 8.0 was employed for statistical analyses and graph drawings. Data are expressed as mean ± SD of a minimum of three independent biological experiments. One-way ANOVA was exploited to evaluate values of experimental data for multiple comparisons with Tukey correction. Differences of P < 0.05 among groups were regarded as statistically significant.

## 3 Results

### 3.1 Exposure to white light caused impaired visual function in mice

A light-induced retinal damage mouse model was developed to examine the therapeutic effects of fullerol. We determined the exposure duration of the mice and selected 15,000 lux white light irradiation for 3 d as a suitable mouse model (Fig. 1a). ERG was used to evaluate functional damage, and representative ERG waveforms are shown in Fig. 1b1–b4. In contrast to the control group, a- and b-waves of the ERGs in the light-exposed groups exhibited a declining trend in amplitude (Fig. 1c1 and c2). The a-wave (58.0 ± 61.62 μV for 3 days (p < 0.001) and 35.44 ± 35.09 μV for 5 days (p < 0.05), respectively) and b-wave amplitudes (152.96 ± 141.84 μV for 3 days (p < 0.05) and 108.53 ± 97.03 μV for 5 days (p < 0.05), respectively) decreased after 3 or 5 d of light exposure compared to those in the control group (142.25 ± 76.06 μV and 280.84 ± 93.57 μV for a- and b-wave amplitudes, respectively) (Fig. 1c1 and c2). However, 1 d of light exposure did not cause significant visual impairment (Fig. 1c1 and c2). To quantify retinal damage, we measured ONL thickness at 200, 400, and 600 μm temporal and nasal sides of the optic nerve head (ONH) (Fig. 1d1–d4). Thickness of ONL was not dramatically different from that of control group, after 1 d of photodamage (Fig. 1e). However, starting from 3 d of light exposure, ONL thickness was inversely proportional to the light exposure time in the light-exposed groups (Fig. 1d5–d8). In addition, we detected ROS generation in the retinas using dihydroethidium (DHE) staining, a cell membrane-permeable fluorescent probe. The DHE staining revealed a noticeable increase in fluorescence intensity throughout the entire retinal thickness in the light-exposed group. Further, the fluorescence intensity exhibited a dramatic and progressive increase with longer exposure time to light (Fig. S2a and b). Thus, we successfully established a light-exposed mouse model.

### 3.2 Fullerol protected retinal structure and function from light damage

To determine whether fullerol exerted protective effects against photic injury, we injected fullerol intravitreally before the retinas were subsequently exposed to 15,000 lux of light. Typical ERG wave shapes are shown in Fig. 2a1–a4. Fig. 2b1 and b2 show the quantization of the spot a- and b-wave ERG amplitudes, respectively. The a-(191.37 ± 28.27 μV for 100 μg/ml, p < 0.001) and b-waves (619.78 ± 107.91 μV for 100 μg/ml, p < 0.001) in the fullerol-pretreated group were evidently higher versus those in the light-exposed group (45.64 ± 18.54 μV and 125.00 ± 48.16 μV for a- and b-wave amplitudes, respectively; p < 0.001) (Fig. 2b1 and b2). However, GSH failed to produce a significant protective effect in the ERG tests on the visual function of light-exposed mice (38.74 ± 5.15 μV for a-wave, 134.87 ± 31.74 μV for b-wave) (Fig. 2b1 and b2). The ONL of the retina treated with fullerol before light exposure was markedly thicker than that of the light-exposed group (Fig. 2c and d). DHE staining showed that fullerol potently quenched most ROS in the retinas (Fig. S3a and b), indicating that fullerol protects the structure and function of the photodamaged retina and provides a better therapeutic outcome than GSH.

### 3.3 Fullerol was accumulated in Müller cell and might enter the nucleus

**Fig. 3:**
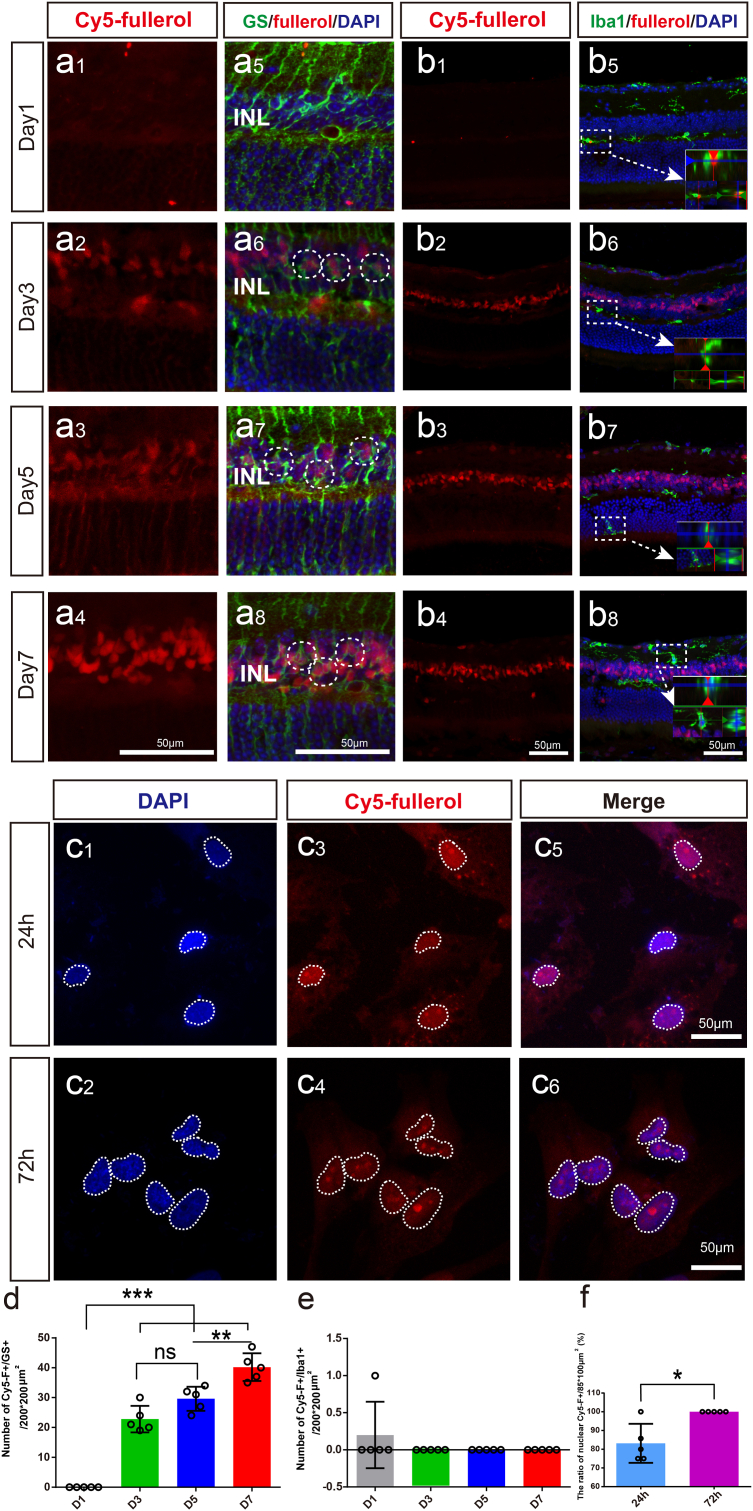
Cy5-fullerol accumulates mainly in the nucleus of Müller glia cells. (a) and (b) Representative confocal photos of glutamine synthetase (GS) and Iba1 immunostaining of retinal cryosections. (c) DAPI staining of MIO-M1 cells. (d) The number of GS-positive and Cy5-fullerol-positive cells. (e) The number of Iba1-positive cells co-localized with Cy5-fullerol. (f) The number of nuclear Cy5-positive cells. Cy5-fullerol/Cy5-F, red fluorescent-labeled fullerol. INL, inner nuclear layer. D1, Cy5-fullerol administration for one day, and so on. The dotted circle outlines co-labeled cells or accumulated fullerol, or cell nuclei. Data are the means ± SD; n = 5. *, P < 0.05; **, P < 0.01; ***, P < 0.001; ns, no significance. Scale bars, 50 μm. (For interpretation of the references to colour in this figure legend, the reader is referred to the Web version of this article.)

To trace the distribution of fullerol in the retina and cells, we used Cy5 red fluorescent-labeled fullerol (Cy5-fullerol) in vivo and in vitro. After injecting Cy5-fullerol into the vitreous cavity, the distribution of Cy5-fullerol was observed in the retinas from day 1–7 (Fig. 3a1–a4). The red fluorescence aggregated in the inner nuclear layer (INL) from day 3–7 and was mainly distributed in the nucleus of the MG (Fig. 3a1–a8). It was confirmed with the co-localization of the MG-specific marker GS and Cy5 fluorescence (Fig. 3a5–a8). The number of co-labeled cells tended to increase over time (Fig. 3d). Iba1 is a cell marker of microglia, only a small fraction of Iba1-positive cells exhibited co-labeling with Cy5-fullerol on the first day (Fig. 3b1 and b5), this co-labeling was not observed at other time points (Fig. 3b2–b8 and e). It indicated that fullerol seemed MG cellular preference and might accumulate within the MG nucleus. To confirm the localization, we supplemented Cy5-fullerol in the culture of the MG cell line MIO-M1 for 24 h and 72 h (Fig. 3c1–c6). Cy5-fullerol was shown to be associated with the nucleus rather than the cytoplasm (Fig. 3c5 and c6). Further, the number of Cy5-fullerol aggregated cells increased over time (Fig. 3f), suggesting that fullerol might mainly act on MG in light-damaged retinas.

### 3.4 Fullerol suppressed the gliotic reaction of Müller cells in the light-damaged retina

**Fig. 4:**
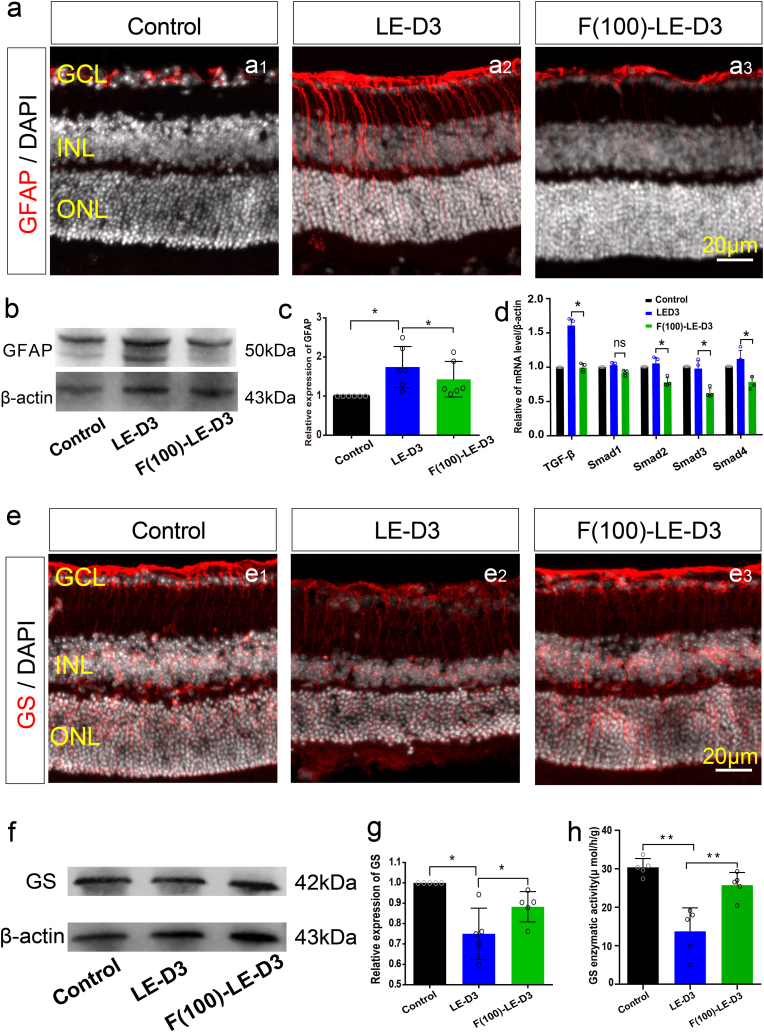
Fullerol pre-treatment alleviates light-induced retinal gliosis reactivity. (a) Representative photomicrographs of glial fibrillary acidic protein (GFAP) immunostaining of retinal cryosections from control, light exposure, fullerol group. (b) and (c) The protein expression of GFAP was measured by western blotting. (d) qPCR analysis of the relative mRNA of TGF-β1, Smad1, Smad2, Smad3 and Smad4. (e) Typical immunofluorescence photos of glutamine synthetase (GS) immunostaining from each group. (f) and (g) The protein expression of GS was measured by western blotting. (h) The detection of GS enzymatic activity. LE-D3, light exposure for three days; F (100)-LE-D3, three days of light exposure after pretreatment with 100 μg/ml fullerol. INL, inner nuclear layer. Data are the means ± SD; n = 4, or 5. *, P < 0.05; **, P < 0.01; ***, P < 0.001; ns, no significance. Scale bars, 20 μm.

Next, we assessed whether fullerol protection against retinal photodamage was associated with the suppression of the gliosis of Müller cells by detecting the protein-levels of glial fibrillary acidic protein (GFAP) and GS, respectively. In control retinas, GFAP immunoreactivity was predominantly distributed in the retinal ganglion cell layer (GCL), where astrocytes are located (Fig. 4a1–a3). Further, GFAP immunoreactivity was markedly increased in the light-damaged mouse retinas compared to control mice, both in the INL and ONL regions, where the soma of Müller cells and the extended process are distributed (Fig. 4a). The immunoreactivity of GFAP in fullerol-pretreated mice was markedly reduced compared to retinal photodamaged mice (Fig. 4a). WB analysis confirmed a noticeable increase in GFAP protein levels in photodamaged retinas (1.54-fold; p < 0.01) versus control retinas (Fig. 4b and c). Fullerol pre-treatment downregulated 17% (p < 0.05) of GFAP protein levels in the retina exposed to light irradiation (Fig. 4b and c). Considering that the TGF-β signaling pathway facilitates MG gliosis [37,38], expressions of TGF-β1, Smad1, Smad2, Smad3, and Smad4 were determined using qPCR. Expect for the expression of Smad1, TGF-β1, Smad2, Smad3, and Smad4 were upregulated in photodamaged retinas, while fullerol downregulated them (Fig. 4d). Moreover, in comparison with undamaged retinas, photodamaged retinas showed a relatively lower immunoreactivity of GS, and this decrease in GS levels was reversed by 22% by fullerol treatment (p < 0.05) (Fig. 4e). The WB data were consistent with findings from immunofluorescence analysis (Fig. 4f and g). Apart from GS content, the enzymatic activity of GS was evaluated, which confirmed that fullerol treatment increased GS activity (25.74 ± 2.95 μ mol/h/g) compared to that of the photodamaged group (13.74 ± 5.45 μ mol/h/g). However, it did not reach the level observed in the control group (30.40 ± 2.04 μ mol/h/g) (Fig. 4h). These findings suggest that fullerol effectively inhibited retinal gliosis during light-induced retinal damage, potentially by modulating the TGF-β signaling pathway.

### 3.5 Fullerol promoted de-differentiation of MG after light damage

**Fig. 5:**
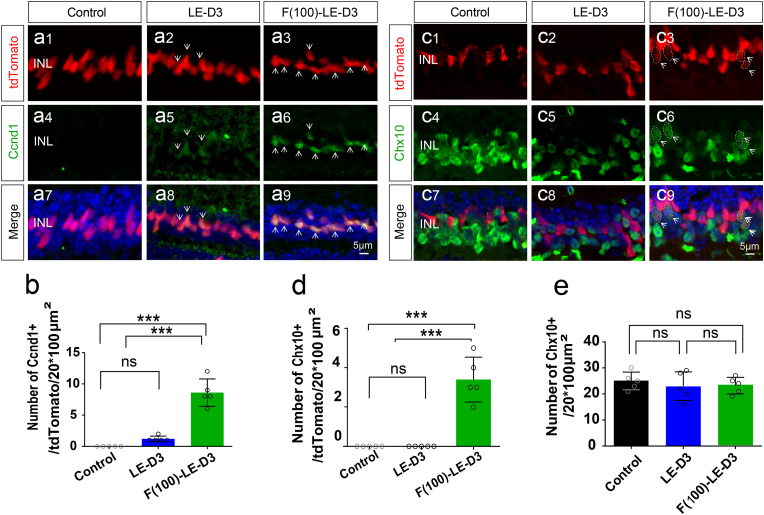
Fullerol pre-treatment promotes proliferation and dedifferentiation of Müller glia cells. (a) and (c) Immunofluorescence labeling for Ccnd1/Chx10 of peripheral areas of control, light-damaged, and fullerol-pretreated retinal sections. (b) and (d) The number of MG (in red) co-localized with Ccnd1/Chx10. (e) The number of single Chx10 positive cells. LE-D3, light exposure for three days; F (100)-LE-D3, three days of light exposure after pretreatment with 100 μg/ml fullerol. Arrow shows positive immunostaining cells. The dotted circle outlines positive cells. Data are the means ± SD; n = 5. ***, P < 0.001; ns, no significance. Scale bars, 5 μm. (For interpretation of the references to colour in this figure legend, the reader is referred to the Web version of this article.)

To investigate whether fullerol promotes MG cell de-differentiation, we established a retinal photodamage model using MG lineage-tracing mice. Cell proliferation was the first step in de-differentiation [39,40], and to determine this, we used the proliferation marker Ccnd1. After light exposure, the number of Ccnd1-positive MG increased in photodamaged retinas (1.2 ± 0.4; p < 0.001) versus control retinas in the non-ciliary margin zone (CMZ) (0 ± 0) (Fig. 5a and b). Fullerol treatment increased the number of Ccnd1-positive MG in the CMZ (21.0 ± 6.16; p < 0.001) (Fig. S4a and b) and non-CMZ retinas (8.6 ± 1.96; p < 0.001) in contrast to the photodamaged group (Fig. 5a and b). To determine whether fullerol treatment induced de-differentiation in MG cells, we detected the retinal progenitor cell marker Chx10 using immunofluorescence staining. No Chx10-positive MG cells were observed in the photodamaged groups (Fig. 5c and d); however, fullerol markedly increased the number of Chx10-positive MG cells in the CMZ (5.0 ± 0.8; p < 0 0.001) (Fig. S4c and d) and non-CMZ groups (3.0 ± 0.5; p < 0.001) (Fig. 5c and d) in contrast to the photodamaged group. As some bipolar cells are also Chx10 positive, we determined the total number of Chx10-positive cells in each group; nevertheless, no significant difference was observed among the groups (Fig. 5e). Additionally, we investigated whether fullerol promoted the stem cell potential of the MG cells in undamaged retinas. Intravitreally injection of fullerol resulted in proliferation of a small population of cells (Fig. S5a and b) and de-differentiation of MG (Fig. S5c and d) 8 d later. Overall, it demonstrates that fullerol promotes MG proliferation and de-differentiation in light-damaged and intact retinas.

### 3.6 Fullerol upregulated the Nrf2 nuclear translocation and the expression of heme oxygenase-1 (HO-1) in the MG cells

**Fig. 6:**
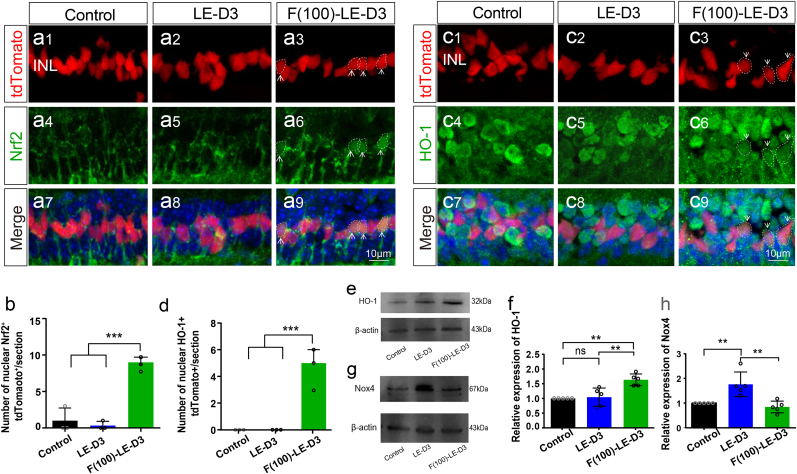
Activation of the Nrf2/HO-1 antioxidant pathway in Müller glia cells with fullerol-pre-treatment in light-induced mouse model. (a) and (c) Immunofluorescence labeling for Nrf2/HO-1 staining of peripheral areas of control, light damaged, and fullerol-pretreated retinal sections. (b) and (d) Number of MG co-localized with Nrf2/HO-1. (e–h) HO-1 and Nox4 protein expression levels were quantified by densitometry and normalized to β-actin levels. LE-D3, light exposure for three days; F (100)-LE-D3, three days of light exposure after pretreatment with 100 μg/ml fullerol. Arrow shows the nuclear import of Nrf2 or HO-1 positive immunostaining cells. The dotted circle outlines positive cells. Data are the means ± SD; n = 5 or 9. **, P < 0.01; ***, P < 0.001; ns, no significance. Scale bars, 10 μm.

Previous studies have demonstrated that fullerol attenuates the damage caused by oxidative stress through activation of the Nrf2/HO-1 signaling pathway [14,41]. Under mild oxidative stress, MG cells exert antioxidant functions through the activation of the Nrf2/HO-1 pathway [24,42]. In the present study, only a minimal amount of Nrf2 entered the nucleus of MG cells (0.3 ± 0.5; p < 0.001) under photodamaged conditions, whereas most Nrf2 was diffusely distributed in the cytoplasm of MG cells (Fig. 6a and b). In the fullerol-treated group, increased Nrf2 was transferred into the nucleus of some MG cells compared to the non-injured group (9.0 ± 0.8 for fullerol pre-treatment group vs. 1.0 ± 1.4 for the control group; p < 0.001) (Fig. 6a and b). HO-1 is a downstream target gene of Nrf2 [43,44]. As shown in Fig. 6c and d, HO-1^+^ MG was markedly increased in the fullerol pre-treatment group (0.3 ± 0.5; p < 0.001) versus the photodamaged group (0.0 ± 0.0). It was further confirmed by WB (Fig. 6e and f). The levels of HO-1 protein were remarkably elevated in the retinas of mice that were pre-treated by fullerol compared to those in the other two groups (Fig. 6f). It suggests that fullerol produces antioxidant effect on photo-induced retinal damage may be mediated through the activation of the Nrf2 pathway.

### 3.7 RNA-seq screened out transcriptions and pathways involved in the protection of fullerol on the photodamaged retina

**Fig. 7:**
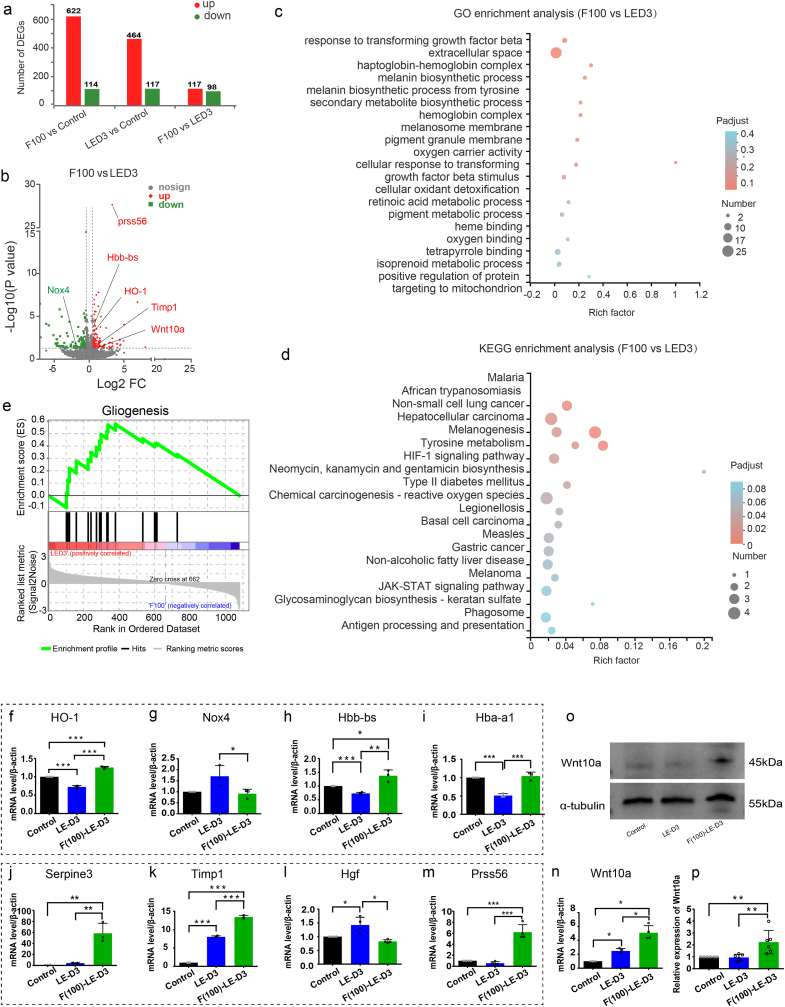
Overall transcriptomic analysis of light stress and fullerol-pre-treatment groups. (a) The number of up-regulation and down-regulation DEGs in each group. Groups are labeled at the bottom. (b)The volcano plot shows DEGs between “F100 vs. LED3”. F100, three days of light exposure after pretreatment with 100 μg/ml fullerol. LED3, three days of light exposure. Each gene is represented by a dot (grey indicates not significant; red, indicates significantly upregulated; green indicates significantly downregulated). (c) and (d) Functional enrichment analysis (GO and KEGG) of the identified DEGs. (e) GSEA plot of regulation of gliogenesis. (f–n) The relative mRNA expression of interested genes. (o) and (p) The protein expression levels of Wnt10a were measured by Western blotting. n = 4 or 6 per group. Data are the means ± SD; *, P < 0.05; **, P < 0.01; ***, P < 0.001. (For interpretation of the references to colour in this figure legend, the reader is referred to the Web version of this article.)

To get a deeper understanding of the function and effect of fullerol, we performed RNA-seq of the retinas of fullerol-pretreated, light-exposed, and photodamaged mice. We determined 215 differentially expressed genes (DEGs) between the light-damaged and the fullerol pre-treatment group, at a conservative threshold of P < 0.05. Furthermore, the volcano plot revealed that 117 genes were up-regulated and 98 were down-regulated among these DEGs (Fig. 7a). DEGs that were up-regulated (Up) or down-regulated (Down) in the photodamaged and fullerol pre-treatment groups are shown in the volcano plot (Fig. 7b). Using differential gene sets built by DEGs for GO terms and KEGG pathway analysis (Fig. 7c and d), we screened MG-related transcription and pathways from the top 20 enriched KEGG and GO pathways.

Gene set variation analysis (GSVA) showed that gliosis-related regulation was evidently reduced in the fullerol pre-treatment group versus photodamaged group (Fig. 7e). The nine DEGs that were primarily related to the regulation of Müller cells, were divided into four categories according to their functions. The first category included genes associated with anti-oxidative stress, including HO-1, hemoglobin alpha, adult chain 1 (Hba-a1), hemoglobin alpha, adult chain 1 (Hba-a2), as well as hemoglobin, beta adult's chain (Hbb-bs), which were elevated in the fullerol pre-treatment group versus photodamaged group. The pro-oxidative gene NADPH oxidase 4 (Nox4) was downregulated in the fullerol pre-treatment group in comparison with the photodamaged group, which was further validated by WB analysis (Fig. 6g and h). The second category included genes related to neuroprotection (anti-inflammatory or inhibition of gliosis), which were up-regulated in the fullerol group, including Serpin peptidase inhibitor3 (Sperine3) and tissue inhibitor of metalloproteinase 1 (Timp1), whereas hepatocyte growth factor (Hgf) was downregulated. Protease serine 56 (Prss56), involved in extracellular matrix remodeling, was up-regulated in the fullerol pre-treatment group. Genes associated with de-differentiation and proliferation, including Wnt10a, were up-regulated in the fullerol group, which was further confirmed by WB (Fig. 7o and p). The screened genes were subjected to RT-qPCR for validation. mRNA levels of anti-oxidative stress related genes, including *HO-1* (Fig. 7f), *Hbb-bs* (Fig. 7h) and *Hba-a1* (Fig. 7i), as well as proliferation and de-differentiation associated genes, such as *Wnt10a,* were remarkably elevated in the fullerol-treated group, whereas the pro-oxidative gene *Nox4* (Fig. 7g), was decreased in the fullerol group, in contrast to that in the photodamaged group (Fig. 7n). Neuroprotection-related genes, including *Sperine3* (Fig. 7j), *Timp1* (Fig. 7k) and *Prss56* (Fig. 7m) were elevated in the fullerol pre-treatment group versus the photodamaged group (Fig. 7f). Consistent with the RNA-seq data, the mRNA level of *Hgf* was obviously reduced in the fullerol pretreated group versus the light-damaged group (Fig. 7l). It suggests that the protective effect of fullerol on the photodamaged retina is a comprehensive process that occurs possibly through increased pathways against oxidative stress, inhibition of matrix remodeling and gliosis, and promotion of MG de-differentiation.

### 3.8 Fullerol alleviated oxidative stress induced by H_2_O_2_ in vitro through Nrf2/HO-1 signaling pathway

**Fig. 8:**
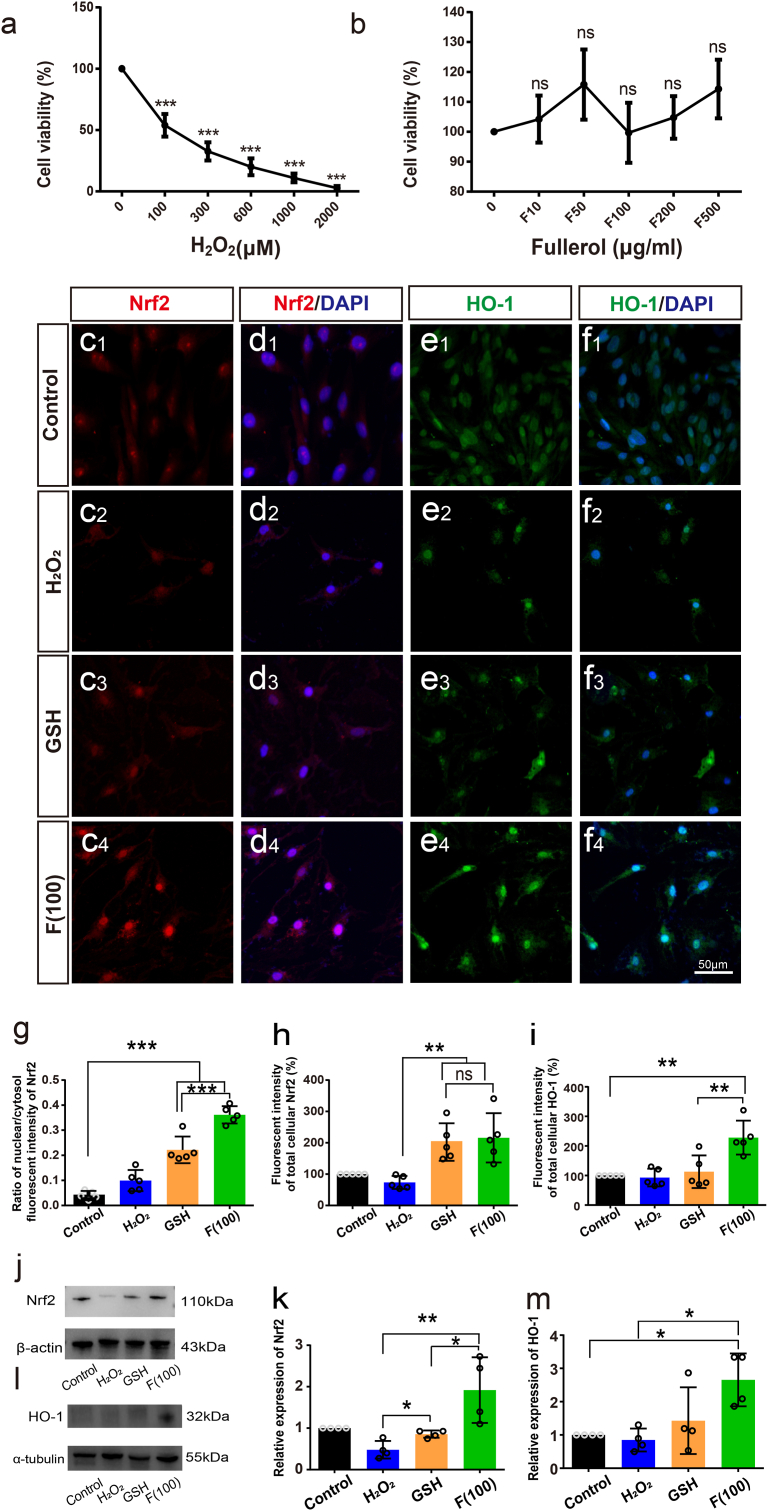
Nrf2 pathway was involved in the fullerol reduced H_2_O_2_-treated oxidative insult in vitro (a) CCK-8 determined the cell viability in MIO-M1 cells after treatment with a concentration gradient of H_2_O_2_ or (b) fullerol. (c–f) Representative fluorescence staining of Nrf2 and HO-1 in MIO-M1 cells. Western blot of Nrf2 and HO-1 proteins in MIO-M1 cells from different treatment groups. (g) Nuclear and cytoplasm of Nrf2 fluorescence ratio in cells. (h) and (i) Quantitative analysis of protein expression of Nrf2 or HO-1 was measured as the integrated fluorescent intensity (per cell). (j–m) The protein expression levels of Nrf2 and HO-1 were measured by Western blotting. F10, pretreatment with 10 μg/ml fullerol; rest in proportion. Control, contains only basic medium; F (100), pretreatment with 100 μg/ml fullerol and then added with 100 μM of H_2_O_2_. GSH, pretreatment with 100 μg/ml of GSH and then added with 100 μM of H_2_O_2_. Data are the means ± SD; n = 4 or 5. *, P < 0.05; **, P < 0.01; ***, P < 0.001; ns, no significance. Scale bar, 50 μm.

To confirm the results in vivo, we treated the human MG cell line MIO-M1 with H_2_O_2_ to create a cell model of oxidative stress. First, we tested the cytotoxic effects of H_2_O_2_ at different concentrations (0–2000 μM) to determine the optimum concentration. Cell viability was lowered dose-dependently (Fig. 8a), where 100 μM of H_2_O_2_ was selected as a suitable concentration and used in subsequent experiments. The cytotoxicity of fullerol was also evaluated. No apparent toxicity was observed despite an increase in fullerol concentration up to 500 μg/ml (Fig. 8b).

Immunofluorescence staining was employed to assess the activation and involvement of the Nrf2 signaling pathway in the fullerol protection (Fig. 8c–f). GSH was used as a positive control. Under physiological conditions, MIO-M1 cells were spindle-shaped, with Nrf2 dispersed in the cytoplasm and only a small amount of Nrf2 dotted in the nucleus (Fig. 8c1 and d1). After exposure to H_2_O_2_, the number of MIO-M1 cells was reduced and they exhibited a circular morphology (Fig. 8c2). Further, Nrf2 entered the nuclei of some MIO-M1 cells (Fig. 8d2). However, treatment with fullerol led to a significant elevation in the number of MG cells and the restoration of normal morphology (Fig. 8c3). Additionally, Nrf2 aggregated in the nucleus of fullerol-treated cells (Fig. 8d3). Nrf2 fluorescent intensity was higher in the fullerol-treated group in comparison to that in the H_2_O_2_-treated group (Fig. 8h), confirming that fullerol treatment markedly increased Nrf2 level in the nucleus while reducing it in the cytoplasm (Fig. 8g). GSH treatment slightly increased the cell number and nuclear aggregation of Nrf2 compared to the H_2_O_2_ -treated group (Fig. 8c4 and d4). The expression of HO-1 was enhanced in the cell cytoplasm and nucleus (Fig. 8e4, f4, and i) after treatment with fulllerol compared to that in the other groups (Fig. 8e1–f3).

Additionally, Western blotting assay demonstrated that the Nrf2 (1.92-fold, p < 0.05) (Fig. 8j and k) and HO-1 (2.66 -fold, p < 0.05) expression levels (Fig. 8l and m) were remarkably elevated in the fullerol-treated group versus in the photodamaged group. Compared with the GSH group, treatment with fullerol raised the Nrf2 (2.24 -fold, p < 0.05) (Fig. 8j and k) and HO-1 (1.86 -fold) expression (Fig. 8l and m). These results indicate that fullerol profoundly activates the Nrf2/HO-1 pathway in MIO-M1 cells and also shows better therapeutic effect than GSH in vitro.

## 4 Discussion

High-intensity exposure to bright white light usually causes oxidative stress and damages the photoreceptors in the retina [35]. Our results showed that fullerol protected the retina from light-induced degeneration. Since fullerol might be preferentially accumulated in MG cells, it probably shifts the cell fate of MG from gliosis to de-differentiation in the photodamaged retina.

### 4.1 The advantages of fullerol

As a carbon nanostructured material, fullerol is easy to be modified by functionalization [45]. After hydroxylation modification, fullerol is characterized with good water solubility and structural stability [46]. Fullerol nanoparticles will not be agglomerated in solution under different pH, temperature, and ionic strength, and their particle size and distribution will not be affected by the microenvironment [47,48]. The aqueous solutions of fullerol do not exhibit significant cytotoxicity even on the solubility limits and demonstrate the safety [49]. The ability of fullerol to scavenge free radicals is not only related to the high affinity of their core to electron donors but also related to their functional groups [50]. Fullerol can quench free radicals through two chemical reaction mechanisms of functional groups, namely carbon addition reaction and hydrogen extraction reaction [51,52], it is quite different from the traditional antioxidants and free radical scavengers.

### 4.2 Fullerol was accumulated in Müller cells preferentially

The entire retina is covered by the MG cells, which are characterized by a radial shape and two stem processes that stretch in opposing directions. Owing to this special anatomy, the MG cells are more sensitive to changes in the microenvironment than microglia, another type of cell in the retina that maintains phagocytic functions [28]. Previous studies have shown that MG cells can phagocytose foreign substances, such as latex beads or intravitreally injected carbon particles [53,54]. Nearly 50% of the MG cells engage in phagocytosis [55]. These observations indicated that MG cells are the primary contributors to phagocytosis.

However, the molecular mechanisms underlying MG phagocytosis remain unknown. Nanoparticle uptake can be separated into two steps: the nanoparticles first bind to the cell membranes either nonspecifically or through ligand-receptor recognition, and are thereafter internalized via the cells through energy-dependent pathways, and are then trafficked to certain subcellular compartments [56]. A previous research suggested that fullerene nanoparticles (NPs) can cross cell membranes by active transport (such as endocytosis) and passive diffusion [57,58]. In endocytosis, NPs first bind to the surface of the cell prior to entering the interior of the cell [59]. Therefore, we assumed that fullerol nanoparticles may be identified by particular ligand-receptor recognition on MG and subsequently adhere to the cell membrane of the MG. However, the specific ligand-receptor recognition of MG requires further research.

### 4.3 Antioxidative stress

Fullerol, a highly efficient and stable antioxidant, typically produces a classical antioxidant effect during light-induced retinal damage. In the present study, using RNA-seq, we identified several important transcription factors associated with oxidative and anti-oxidative stress, including *Nox4, Hbb-bs, Hb-a1, Hb-a2*, and *HO-1*. *Nox4* is a major gene involved in light-induced retinal damage. It is one of the major isoforms of cytosolic nicotinamide adenine dinucleotide phosphate oxidase that modulates ROS production and contributes to pathological retinal angiogenesis [60,61]. We showed that fullerol inhibited the increase in *Nox4* expression in retinas induced by light exposure, which was further verified by WB analysis. Hemoglobins, including *Hbb-bs, Hb-a1*, and *Hb-a2*, are upregulated in fullerol-treated photodamaged retinas. Hbs are localized in the inner mitochondrial membrane of neurons and glia and exert an essential role in maintaining normal mitochondrial function by stabilizing their membranes [62]. The elevated expression of Hbs by fullerol may protect cells from oxidative stress by maintaining mitochondrial membrane stability.

Nrf2, as a canonical endogenous antioxidant response pathway, serves a pivotal role in the protection of cells from oxidative damage by upregulating the expression of cytoprotective enzymes, such as HO-1. Activation of Nrf2 evokes a comprehensive array of cellular defenses against endogenous and exogenous stressors. Nrf2 is commonly inactivated through binding to Kelch-like ECH-associated protein 1 (KEAP1) in the cytoplasm. Under oxidative stress, nonetheless, Nrf2 dissociates from KEAP1 and accumulates in the nucleus to perform its antioxidant functions by modulating the expression of associated genes [63,64]. The Nrf2 pathway is pivotal for maintaining redox homeostasis in MG [24,31]. We confirmed that fullerol upregulated Nrf2 expression and its target gene, HO-1, in the MG cells upon nuclear translocation in vivo and in vitro. Elevated protein levels of HO-1 were further verified by WB analysis. As stem cell fate is modulated by metabolic homeostasis and cellular redox processes, Nrf2 plays a vital role in modulating survival, stem cell quiescence, proliferation, self-renewal, differentiation and dedifferentiation [32,33,65]. Additionally, fullerol effectively inhibited ROS production and created a favorable microenvironment for the survival of bone marrow mesenchymal stem cells [21]. Therefore, we speculated that fullerol might regulate the retinal redox microenvironment through the Nrf2 pathway and thus promote MG de-differentiation.

### 4.4 Suppressing gliosis and remodeling extracellular matrix (ECM)

Continuous injury often leads to retinal gliosis. MG cells respond to non-specific and specific reactions and play a pivotal role in gliosis. Unspecific responses are characterized by somatic hypertrophy (swelling), proliferation, and the upregulation of intermediate filaments, including vimentin, nestin, and GFAP. GFAP is a sensitive marker for gliosis [66]. The present study indicates that fullerol considerably inhibits reactive gliosis in the photodamaged retina, as indicated by decreased GFAP expression. In addition, the specific response of the MG cells to neuronal injury involves the downregulation of GS due to the loss of numerous photoreceptors in the photodamaged retina, or retinitis pigmentosa [66,67]. GS is typically expressed in MG cells [66,67]. Enhanced glutamate metabolism due to increased GS enzyme activity may protect retinal neurons [68,69]. We demonstrated that GS expression and activity are downregulated in photodamaged retinas and upregulated in fullerol-treated retinas. Previous studies have indicated that the TGF-β pathway promoted MG gliosis in retinal injury and was regarded as a target for anti-gliosis therapy [37,70]. However, the present study confirms that fullerol inhibits the TGF-β signaling pathway by reducing the production of endogenous TGF-β1 and Smad2/3/4. Thus, fullerol can markedly inhibit specific and non-specific glial responses induced by light exposure, primarily through the TGF-β pathway.

The ECM is pivotal for maintaining retinal structure, and ECM remodeling is often detrimental to the retina [71,72]. Timp1 inhibits matrix remodeling by downregulating matrix metalloproteinases (MMPs) production [73]. The hepatocyte growth factor is expressed in the MG and interacts with ECM proteins and MMPs in proliferative retinal disorders [74,75]. Prss56 is expressed by late retinal progenitor cells, and MG encodes a trypsin-like serine protease [76,77]. This demonstrates that fullerol increases Prss56 and Timp1 expression while decreasing HGF expression, indicating that fullerol influences the ECM remodeling to improve the retinal microenvironment.

### 4.5 Activation of the stem cell potential of MG

We further investigated whether the inhibition of gliosis by fullerol would change the fate of MG cells to reprogram them into stem-like cells. Ccnd1 is a specific cyclin that regulates the G1/S phase [78,79] and potentially promotes the progenitor-like identity of MG cells [80,81]. We showed that fullerol increased the number of MG cells that entered the cell cycle (Ccnd1-labeled MG) during light-induced retinal degeneration. However, light damage alone activated only a small amount of MG proliferation. This suggests that fullerol initiates the shift of MG cells to a proliferative progenitor-like state. It is consistent with a previous report that states overexpression of β-catenin (a transcriptional effector of the Wnt pathway) in an adult mouse could promote the re-entry of MG cells into the cell-cycle in uninjured retinas [82]. Moreover, fullerol increased the ratio of Chx10-positive cells among MG cells in CMZ and non-CMZ in the photodamaged retinas of mice. As Chx10 is expressed both in retinal progenitors and bipolar cells in the retina, we used Glast-CreER transgenic mice crossed with the Cre-inducible CAG-LSL-tdTomato reporter in the present study, in which the MG cells were specifically labeled with tdTomato. Fullerol treatment increased the ratio of Chx10/tdTomato double-labeled progenitors among MG cells in the CMZ and non-CMZ of the retina compared to the photodamaged group. This finding implies that fullerol enables MG cells to enter into a progenitor state in the injured or non-injured retina, which may protect the retina. Activation of Wnt signaling has been proven to facilitate the proliferation of MG cells and neurogenic potential in adult mouse retinas, which helps regenerate the injured retina [83,84]. We confirmed that Wnt10a, a Wnt ligand and an activator of Wnt signaling, was up-regulated after fullerol pre-treatment compared to the photodamaged group. Further, CyclinD1 (the gene name of Ccnd1) is a target gene of the Wnt signaling pathway [85,86]. The number of Ccnd1-positive MG dramatically elevated in the fullerol group, implying that fullerol promoted MG cell de-differentiation, possibly through modulating the Wnt pathway.

### 4.6 Crosstalk between TGF-β and the Wnt signaling pathways

Crosstalk between Wnt signaling and TGF-β is widely investigated in early embryonic development, morphogenesis, and tumor progression [87]. TGF-β can reduce cellular β-catenin levels in various cell types [88], which inhibits midbrain development in mouse embryos [89]. It has demonstrated that MG in mammalian retinas express both the receptors and ligands of TGF-β and Wnt signaling [[90], [91], [92]]. The canonical Wnt signaling pathway acts an essential role in the proliferation and differentiation of MG-derived progenitors [90]. Growth factors inducing photoreceptor differentiation have been shown to upregulate canonical Wnt signaling components in human MG [93], which was inhibited by TGF-β [94]. Further research is needed to determine how fullerol modulates TGF and TGF-β signaling and how they interact with one each.

### 4.7 Limitations

The pharmacokinetics, absorption, and local effective doses of fullerol have not been extensively studied. However, in a study using radiolabeling modified fullerenes, 24 h after intraperitoneal injection of fullerenes, the distribution of fullerenes was detected in blood, heart, lung, liver, stomach, intestine, bone, muscle, spleen and kidney, and its main route of excretion was through the kidneys [95]. In a different investigation, fullerenes were nose-only inhalation for 90 days, fullerenes residues were found in lung tissue as well as in liver and spleen tissue [96]. These studies indicate that fullerenes are widely distributed in organisms and that organisms have a certain metabolic clearance capacity for fullerenes. And the delivery method affects the biodistribution and metabolism of fullerenes.

The pharmacokinetics of fullerol in human ocular organ function has not yet been investigated. The fullerol employed in this work is a fullerene derivative that has undergone hydroxylation, making it extremely water-soluble, stable, and capable of penetrating biological barriers like the blood-retinal barrier as well as cell membranes [97,98]. Our research proved that with a single intravitreal injection into the vitreous cavity in a photodamaged mouse model, the fluorescence was maintained and observed in the retina on the 7th day after the injection. Furthermore, our prior findings demonstrated that fullerol kept the protective effect on the retina on the 17th day after intravitreal injection in rd1 mice [99]. Other studies have shown that fullerol has a half-life of up to 26 days [100]. It has been reported that nanoparticles can be eliminated from the vitreous body by crossing the blood-retinal barrier posteriorly or reaching the posterior chamber anteriorly [101]. For fullerol was prepared as a mixture and the amount of aqueous humor in mice was too small, it was difficult to isolate it from the vitreous and accurately quantify its concentration in vivo. Given the current results, we speculate that fullerol has a longer protective time for the damaged retina.

### 4.8 Clinical perspectives

Our findings highlight the therapeutic potential of fullerol and its ability to modulate MG behavior, providing valuable insights into the development of new strategies for treating retinal diseases. Our research expands the understanding of antioxidants and their application in addressing light-induced retinal damage, providing a latent impact on clinical practice and future research in ophthalmology. However, the safety and durability of fullerol in the treatment of retinal diseases are still urgent issues to be resolved.

In summary, we demonstrated that fullerol mainly acts on the MG cells in the photodamaged retina and shifts the cell fate of MG from gliosis to de-differentiation. However, we failed to clarify whether MG-derived progenitors further differentiated into photoreceptors damaged by high-intensity light exposure.

## 5 Conclusions

Light exposure caused retinal degeneration, reduced visual function, and increased gliotic responses, whereas fullerol reversed these effects in vivo and in vitro. Fullerol might decrease the level of oxidative stress in light-induced mouse models and promote the de-differentiation potential of MG into endogenous stem cells. Our study offers evidence for the utilization of fullerol in the treatment of retinal diseases.

## Author contributions

Conceptualization: HWX, ZT, ZC.

Methodology: ZC, ZYY, LLG, LDA, JLY, MRZ, SZ.

Investigation: HWX, ZC, ZT.

Visualization: ZC, ZYY, XNH, HG, JLY.

Supervision: HWX, ZT, ZJG.

Writing—original draft: ZC.

Writing—review & editing: HWX, ZC, ZT, JCH, ZC, ZF, LYM.

## Declaration of competing interest

The authors declare that they have no known competing financial interests or personal relationships that could have appeared to influence the work reported in this paper.

## Data Availability

Data will be made available on request.
