# Supplementary material for: Fullerol rescues the light-induced retinal damage by modulating Müller glia cell fate
Source: Redox Biol. 2023 Oct 5;67:102911. doi: 10.1016/j.redox.2023.102911 (PMC10570010; doi:10.1016/j.redox.2023.102911)
Supplement: Multimedia component 1 [file mmc1.docx]

**Supplementary material for**

**Fullerol rescues the light-induced retinal damage by modulating Müller glia cell fate**

**Supplementary figures**


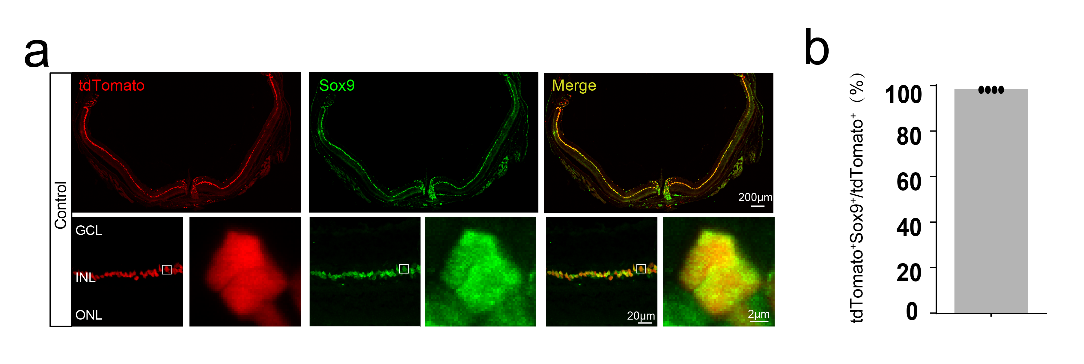


**Fig. S1 Validation of labeling efficiency in Müller glial (MG) lineage-tracing mouse.**

(a) Showing retina immunolabeled for Sox9 in MG lineage-tracing mouse (Sox9, in green). (b) The co-labeling rate of Sox9 positive and tdTomato. ONL, outer nuclear layer; INL, inner nuclear layer; GCL, ganglion cell layer. Scale bar, 20 or 200 μm.


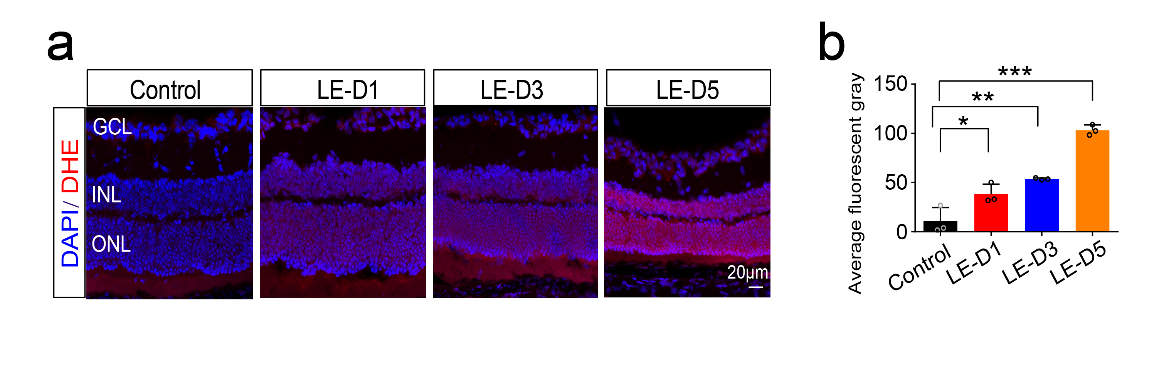


**Fig. S2 Light-induced oxidative stress in the retina of mice.**

(a) DHE staining of retinas of several time points after light insult. (b) Quantification of DHE fluorescence. LE-D1, light exposure for one day; LE-D3, light exposure for three days; LE-D5, light exposure for five days; F(100)-LE-D3, three days of light exposure after pretreatment with 100 μg/ml fullerol. ONL, outer nuclear layer; INL, inner nuclear layer; GCL, ganglion cell layer. Data are the means± SD; n =6. *, P < 0.05; **, P < 0.01; ***, P < 0.001. Scale bar, 20 μm.


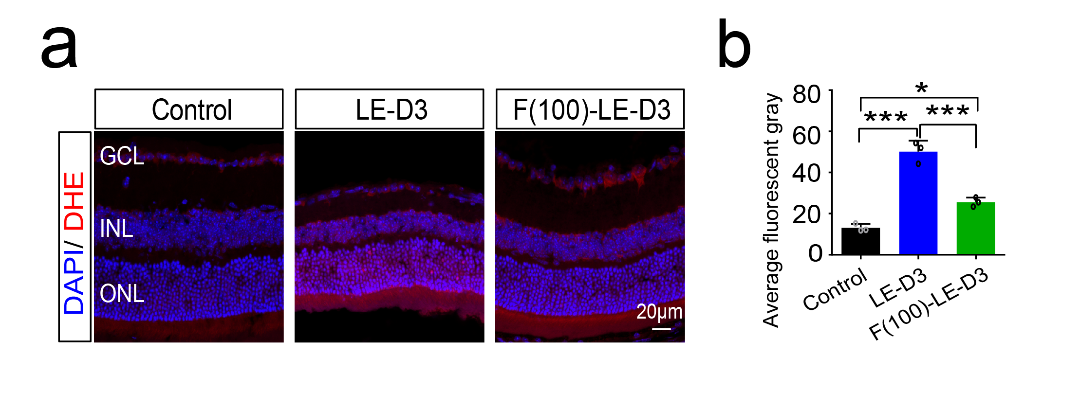


**Fig. S3 Fullerol relieves light-induced oxidative stress in the mice.**

(a) DHE staining of retinas from the control group, light-induced group, and fullerol-pretreatment group. (b) Quantification of DHE fluorescence. LE-D3, light exposure for three days; F(100)-LE-D3, three days of light exposure after pretreatment with 100 μg/ml fullerol. ONL, outer nuclear layer; INL, inner nuclear layer; GCL, ganglion cell layer. Data are the means± SD; n =6. *, P < 0.05; ***, P < 0.001. Scale bar, 20 μm.


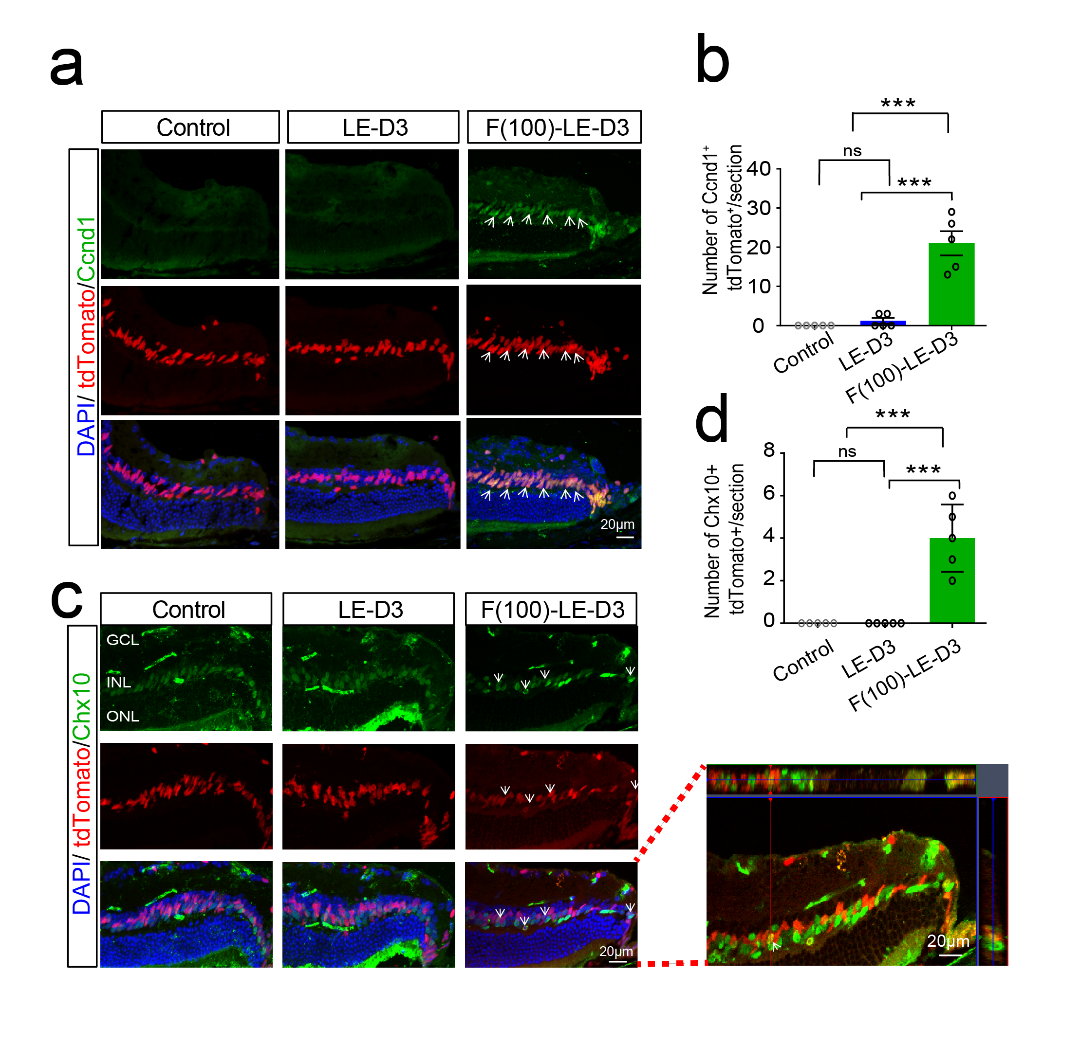


**Fig. S4 Fullerol promotes the proliferation and dedifferentiation of MGs in the ciliary margin zone (CMZ) of mice.**

(a) and (c) Immunofluorescence labeling for Ccnd1/Chx10 of CMZ of control, light damaged, and fullerol-pretreated retinal sections. (b) and (d) Number of MGs (Sox9 positive cells) co-localized with Ccnd1/Chx10. There is an orthogonal diagram presented in (c). LE-D3, light exposure for three days; F(100)-LE-D3, three days of light exposure after pretreatment with 100 μg/ml fullerol. The arrow shows immunostaining positive cells. Data are the means± SD; n =5. ***, P < 0.001; ns, no significance. Scale bar, 20 μm


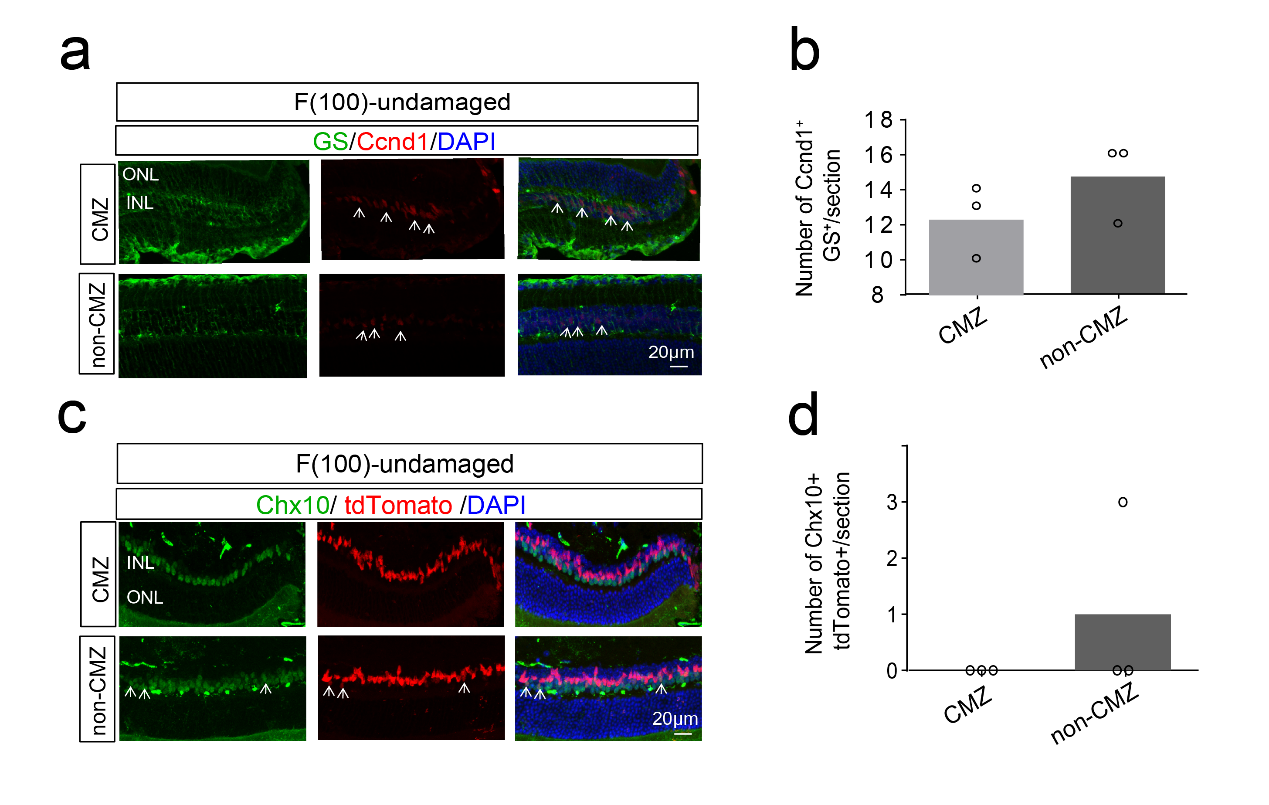


**Fig. S5 Fullerol promotes the proliferation and dedifferentiation of MGs in various regions in the undamaged retinas of mice.**

(a) and (c) Immunofluorescence labeling for Ccnd1(GS) /Chx10 of CMZ and non-CMZ of undamaged retinal sections. (b) Number of GS co-localized with Ccnd1 in CMZ and non-CMZ. (d) Number of MGs (in red) co-localized with Chx10 in CMZ and non-CMZ. Data are the means± SD; n =6. Scale bar, 20 μm.


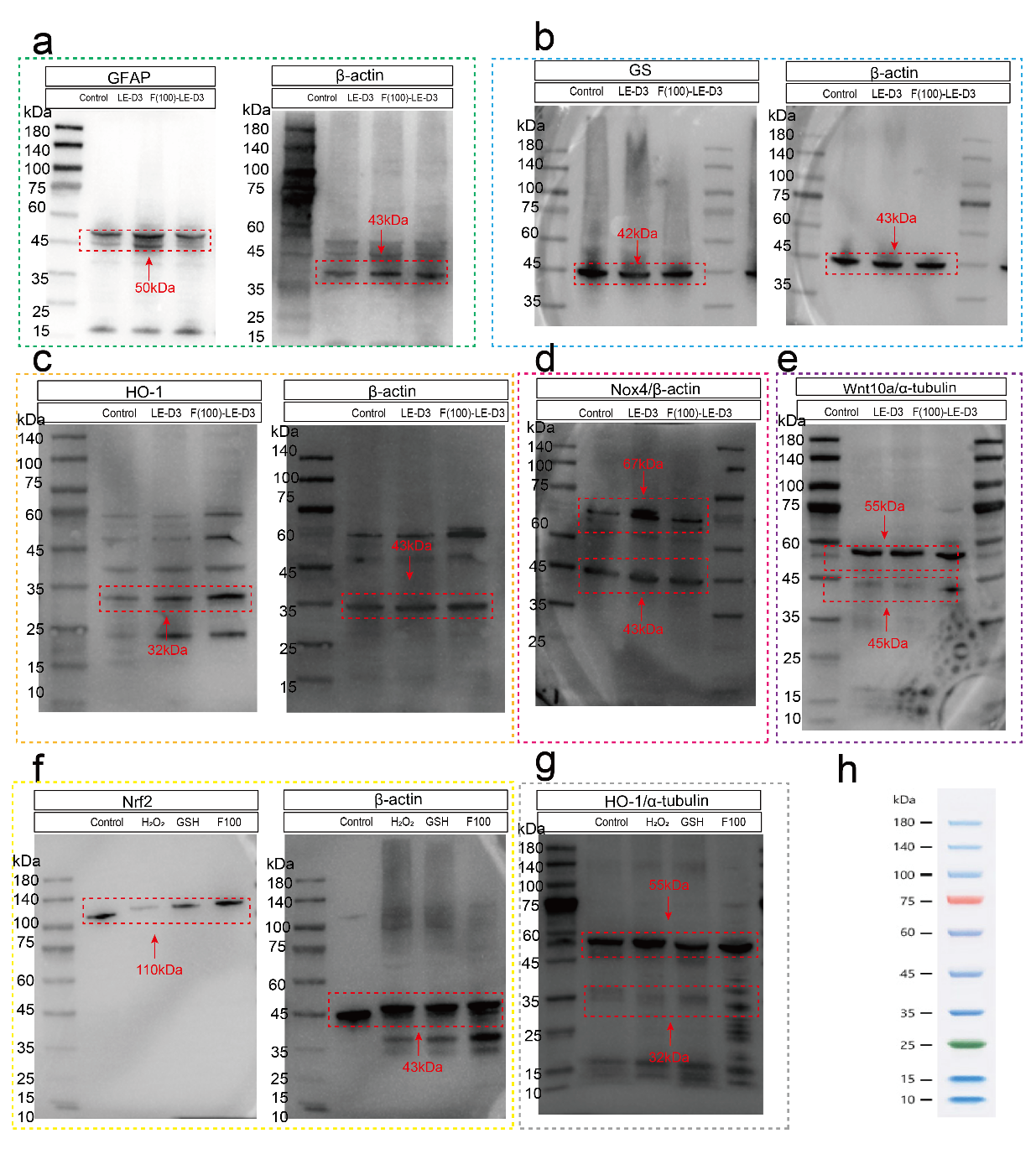


**Fig. S6 Intact images of Western blotting.**

(a) The images of protein bands of GFAP and its reference protein β-actin. (b) The images of protein bands of GS and its reference protein β-actin. (c) The images of protein bands of HO-1 and its reference protein β-actin. (d) The image of protein bands of Nox4 and β-actin. (e) The image of protein bands of Wnt10a and its reference protein α-tubulin. (f) The images of protein bands of Nrf2 and its reference protein β-actin (MIO-M1 cells). (g) The image of protein bands of HO-1 and its reference protein α-tubulin (MIO-M1 cells). (h) The image of bands of color mixed protein marker.

**Supplementary tables**

**Table 1. Summary of primary and secondary antibodies**

| Antibody name | Source | Catalog number | Dilution ratio |
| --- | --- | --- | --- |
| Glial fibrillary protein (GFAP) | DAKO | Z033401 | 1:500 (IF);  1:1000 (WB) |
| Glutamine synthetase (GS) | ABclonal | A19641 | 1:500 (IF);  1:1000 (WB) |
| β-actin | Immunoway | YT0099 | 1:1000 (WB) |
| α-tubulin | Beyotime | AF0001 | 1:1000(WB) |
| Sox9 | Immunoway/ Abcam | YM6546/ ab185966 | 1:500 (IF) |
| Iba1 | Wako | 019-19741 | 1:400(IF) |
| CyclinD1 | ABclonal | A19038 | 1:200 (IF) |
| Chx10 | Santa Cruz | SC-373151 | 1:500(IF) |
| Nrf2 | Immunoway | YT3189 | 1:200 (IF)/ 1:1000 (WB) |
| Wnt10a | Immunoway | YN0283 | 1:500 (WB) |
| Heme Oxygenase1 (HO-1) | Proteintech | 10701-1-AP | 1:50 (IF); |
| Heme Oxygenase1 (HO-1) | ABclonal | A19062 | 1:1000 (WB) |
| 488 donkey-mouse | Invitrogen | A21202 | 1:500 |
| 568 donkey-rabbit | Invitrogen | A10042 | 1:500 |
| 488 donkey-rabbit | Invitrogen | A21206 | 1:500 |
| HRP-Goat anti-rabbit | Beyotime | A0208 | 1:2000 |
| HRP-Goat anti-mouse | Beyotime | A0216 | 1:2000 |

**Table 2. Primer sequences**

| Gene name | Forward 5’-3’ | Reverse 5’-3’ |
| --- | --- | --- |
| TGF-β1 | TGGAGCAACATGTGGAACTC | GTCAGCAGCCGGTTACCA |
| Smad1 | GGCGACATATTGGGAAAGGA | TCACTGAGGCATTCCGCATA |
| Smad2 | TCACTGAGGCATTCCGCATA | AAGCCATCACCACTCAGAATTG |
| Smad3 | CACTGATCTACCGTATTTGCTGT | CACGCAGAACGTGAACACC |
| Smad4 | GGCAGTAGATAACGTGAGGGA | ACACCAACAAGTAACGATGCC |
| β-actin | GCAAAGGTTTCACTTTCCCCA | AAGTCCCTCACCCTCCCAAAAG |
| Wnt10a | GGTCAGCACCCAACGACATCC | TGGCGTAGGCGAAAGCACTCT |
| Timp1 | AGTCCCAGAACCGCAGTGAA | GTGGCAGGCAAGCAAAGTGA |
| HGF | CAAATGCAAGGACCTTAGAG | CCAGAAGATATGACGGTGTAA |
| Hbb-bs | AAAGGTGAACGCCGATGAAG | ATGATAGCAGAGGCAGAGGATAG |
| Hba-a1 | CAGGTCAAGGGTCACGGCAAGA | GGGTGAAATCGGCAGGGTGG |
| Hmox1 | TGACAGAAGAGGCTAAGACCG | AGTGAGGACCCACTGGAGGA |
| Serpine3 | ACGGCACAACTCCAGTCAAG | CCTCCGTGGTAGTGCTGTTAG |
| Nox4 | TCAAACAGATGGGATTCAGA | GAGTTGTTCCGGTTACTCAA |
| Prss56 | GAGGCTGCAACTTGGAGGGT | GTCTGCGGGTCAAACTTAGGG |
